# Resection and Primary Repair of an Arteriovenous Fistula Aneurysm in a 29-Year-Old Female Patient With Systemic Lupus Erythematosus: A Case Report

**DOI:** 10.7759/cureus.95673

**Published:** 2025-10-29

**Authors:** Jorge A Garcia Garza, Rafael Duran-González, J Sinhue Arguelles-Ambriz, Riky Luis Perez-Lucas, Edgar A Rodriguez Treviño, Ivan E Murillo-Barrios, Bianca S Garcia Beattie

**Affiliations:** 1 General Surgery, Hospital Regional de Monterrey – ISSSTE (Instituto de Seguridad y Servicios Sociales de los Trabajadores del Estado), Monterrey, MEX; 2 Vascular Surgery, Hospital Dr Manuel Cardenas de la Vega – ISSSTE (Instituto de Seguridad y Servicios Sociales de los Trabajadores del Estado), Culiacan, MEX; 3 Vascular Surgery, Hospital Regional de Monterrey – ISSSTE (Instituto de Seguridad y Servicios Sociales de los Trabajadores del Estado), Monterrey, MEX; 4 General Surgery, Vicerrectoria de Ciencias de la Salud, Universidad de Monterrey, Monterrey, MEX

**Keywords:** aneurysmal arteriovenous fistulas, arteriovenous (av) fistula, arteriovenous fistula repair, vascular repair, vein aneurysm

## Abstract

Arteriovenous fistulas (AVFs) are a common vascular access method for patients with end-stage renal disease requiring hemodialysis, but they can develop complications such as aneurysms, which may compromise access function and patient comfort. We report the case of a 29-year-old female patient with systemic lupus erythematosus (SLE) and KDIGO (Kidney Disease: Improving Global Outcomes) V chronic kidney disease who developed a painful aneurysm in the venous segment of a brachiocephalic AVF. Clinical evaluation revealed a dilated, erythematous, and tender venous segment without thrill. The patient underwent surgical resection of the aneurysmal segment and primary end-to-end repair, using a Nelaton probe for internal support and reinforcement with fibrin sealant. Postoperative recovery was favorable, with complete symptom resolution and adequate flow confirmed on follow-up ultrasound two months later. This case illustrates the effectiveness of surgical management with primary repair in selected patients and highlights the need for individualized treatment strategies, particularly in those with risk factors such as immunosuppressive therapy. Further research comparing surgical and endovascular approaches is warranted to optimize outcomes in AVF-related aneurysms.

## Introduction

Hemodialysis is a renal replacement therapy indicated for patients with end-stage renal disease in whom residual kidney function is insufficient to maintain metabolic and fluid balance. An adequate vascular access is essential to ensure effective hemodialysis, and current options include central venous catheters, arteriovenous grafts (AVG), and arteriovenous fistulas (AVF), with the latter being the preferred method due to superior long-term patency and lower rates of infection and thrombosis [1]. Despite these advantages, AVFs are not exempt from complications; approximately one-third of patients develop access-related issues such as stenosis, thrombosis, infection, or aneurysmal degeneration. It is important to note that not all AVF-associated aneurysms require intervention-small, stable aneurysms without rapid progression or complications may be managed conservatively under structured surveillance. However, enlarged or symptomatic aneurysms carry a risk of rupture, bleeding, skin necrosis, or access failure and therefore may require surgical or endovascular repair [1-3]. We present the case of a 29-year-old female patient with systemic lupus erythematosus (SLE) who developed venous aneurysms in her AVF, successfully managed through aneurysm resection and primary repair.

## Case presentation

**Figure 1:**
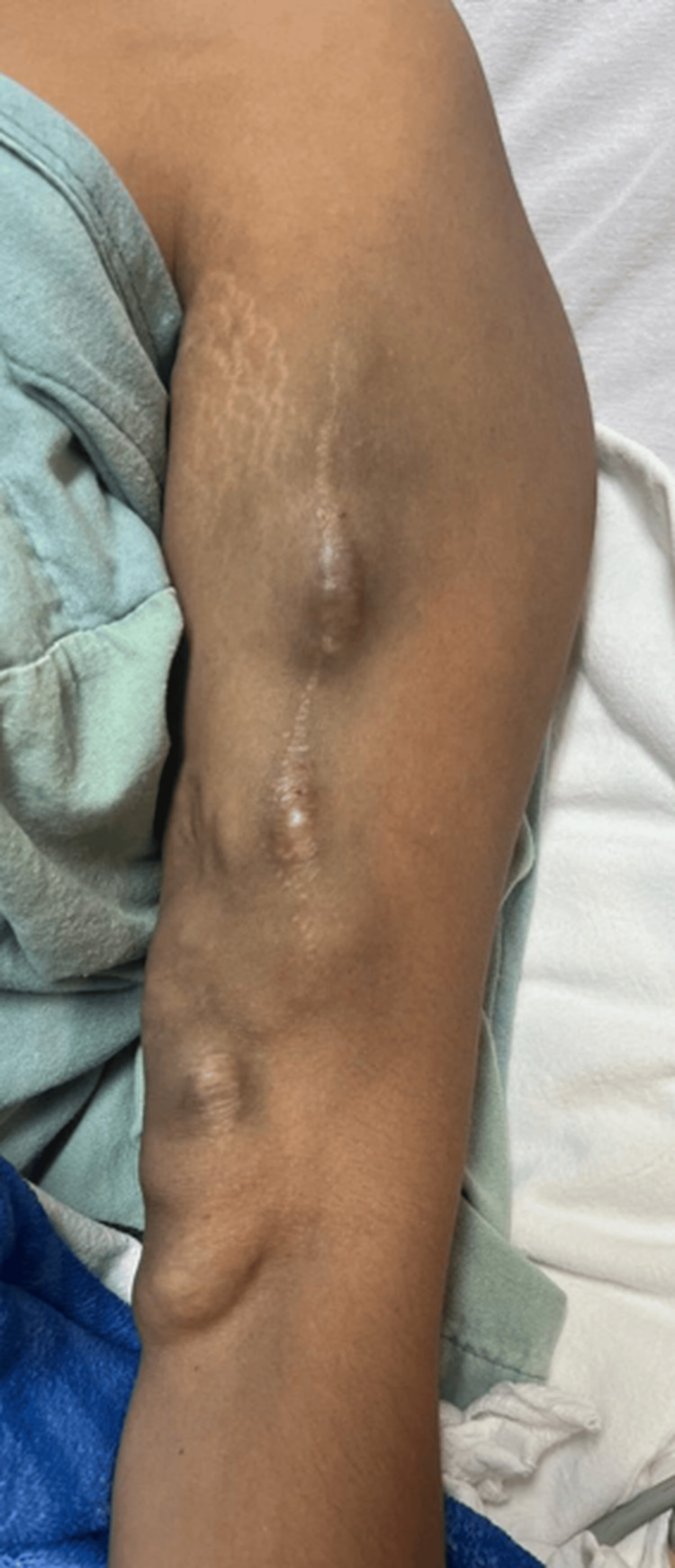
Preoperative image Image shows aneurysms in the venous segment of the arteriovenous fistula in the left arm of the patient.

A 29-year-old woman with a history of SLE, secondary arterial hypertension, and KDIGO (Kidney Disease: Improving Global Outcomes) V [4] kidney disease, who had previously been hospitalized for ulnar arterial steal syndrome related to an AVF, has undergone a new brachiocephalic AVF created two years ago. The patient reported experiencing spontaneous pain episodes and an increase in venous segment volume over the past two years without affecting her hemodialysis. Intense pain occurred during hemodialysis sessions three months before her admission, followed by AVF dysfunction, prompting evaluation by our angiology service. The evaluation revealed venous segment dilation in the bicipital region, extending to the ipsilateral axillary region (Figure 1). This area was painful at rest and on palpation, erythematous, and without a thrill. The patient was protocolized for AVF treatment.

**Figure 2:**
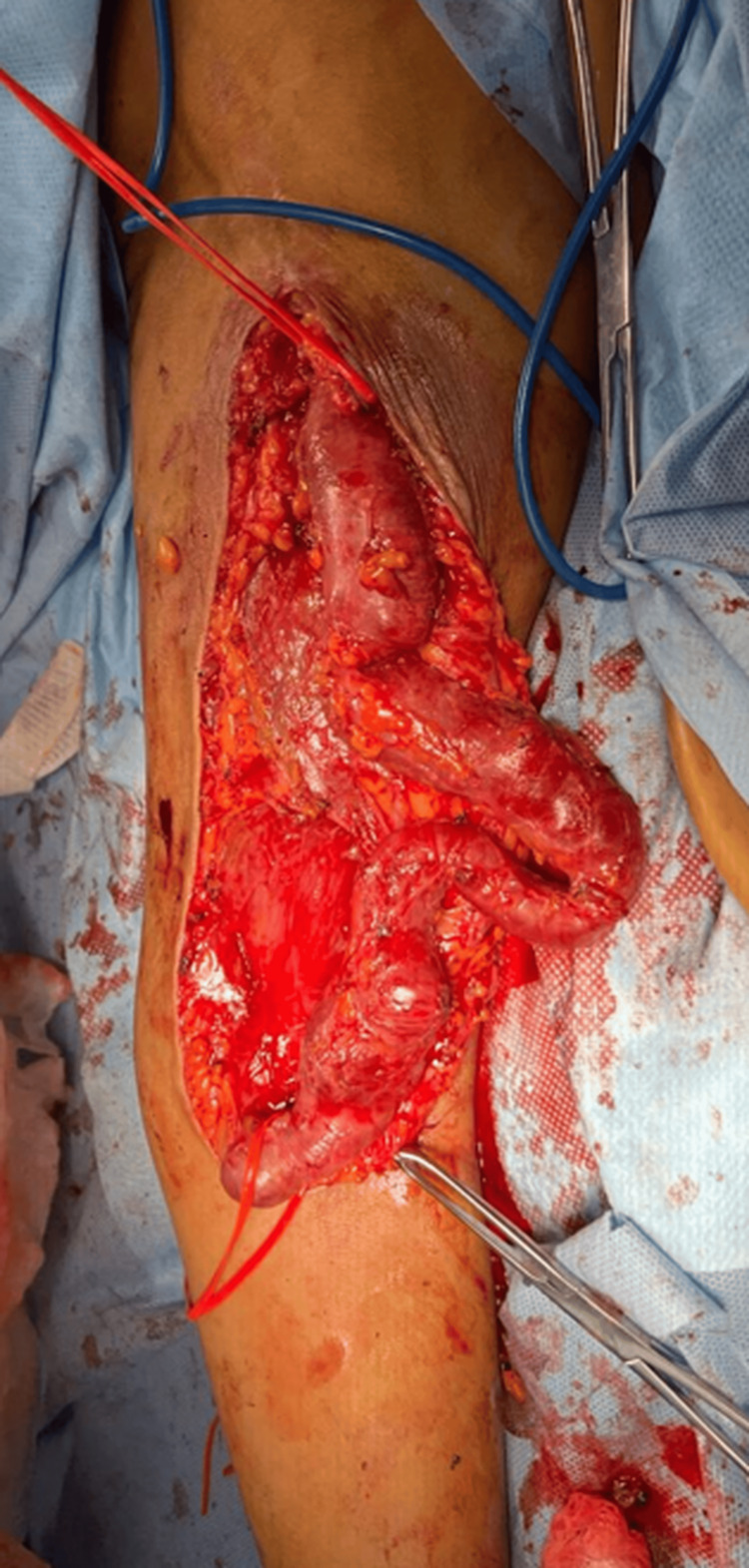
Aneurysms in the venous segment Complete release of the venous segment of the arteriovenous fistula shows tortuosities along the vein.

An incision was made along the entire affected venous segment of the AVF. The venous segment was dissected until it was fully released, and its tributary veins were ligated (Figure 2). The brachial artery and cephalic vein were observed extending down to the infraclavicular area, and neither was dilated. The cephalic vein was completely resected after achieving proximal and distal vascular control.

**Figure 3:**
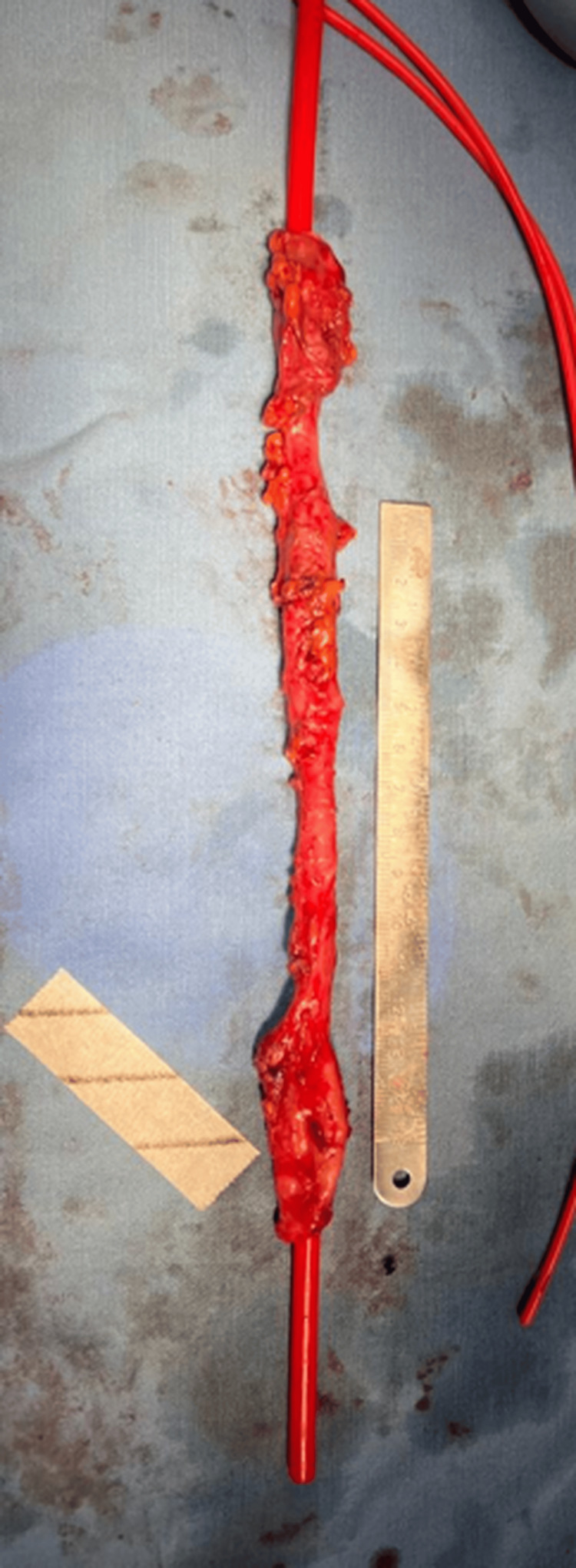
Nelaton Venous segment with Nelaton catheter for support.

**Figure 4:**
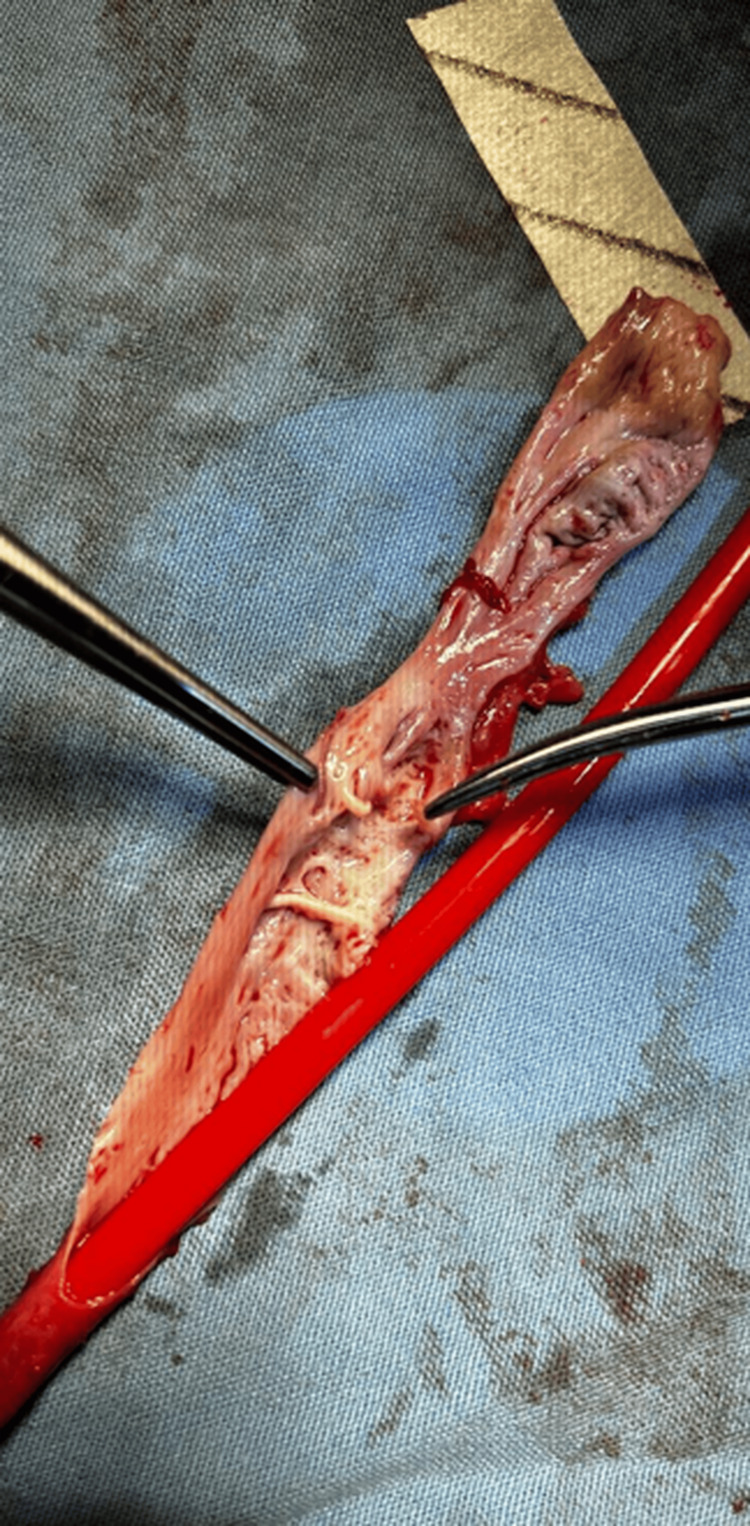
Synechiaes Synechiaes found along the intima of the open venous segment, probably because of chronic turbulent flow and endothelial injury.

Initially, a Nelaton probe was placed inside the aneurysmal vein (Figure 3), and the anterior wall of the vein was resected to remove the aneurysmatic portions. Inside it, synechiae were observed and cut (Figure 4). Continuous support was provided in the cephalic vein using a Nelaton probe as a mould, and it is secured with hemostatic stitches to achieve a tight closure.

**Figure 5:**
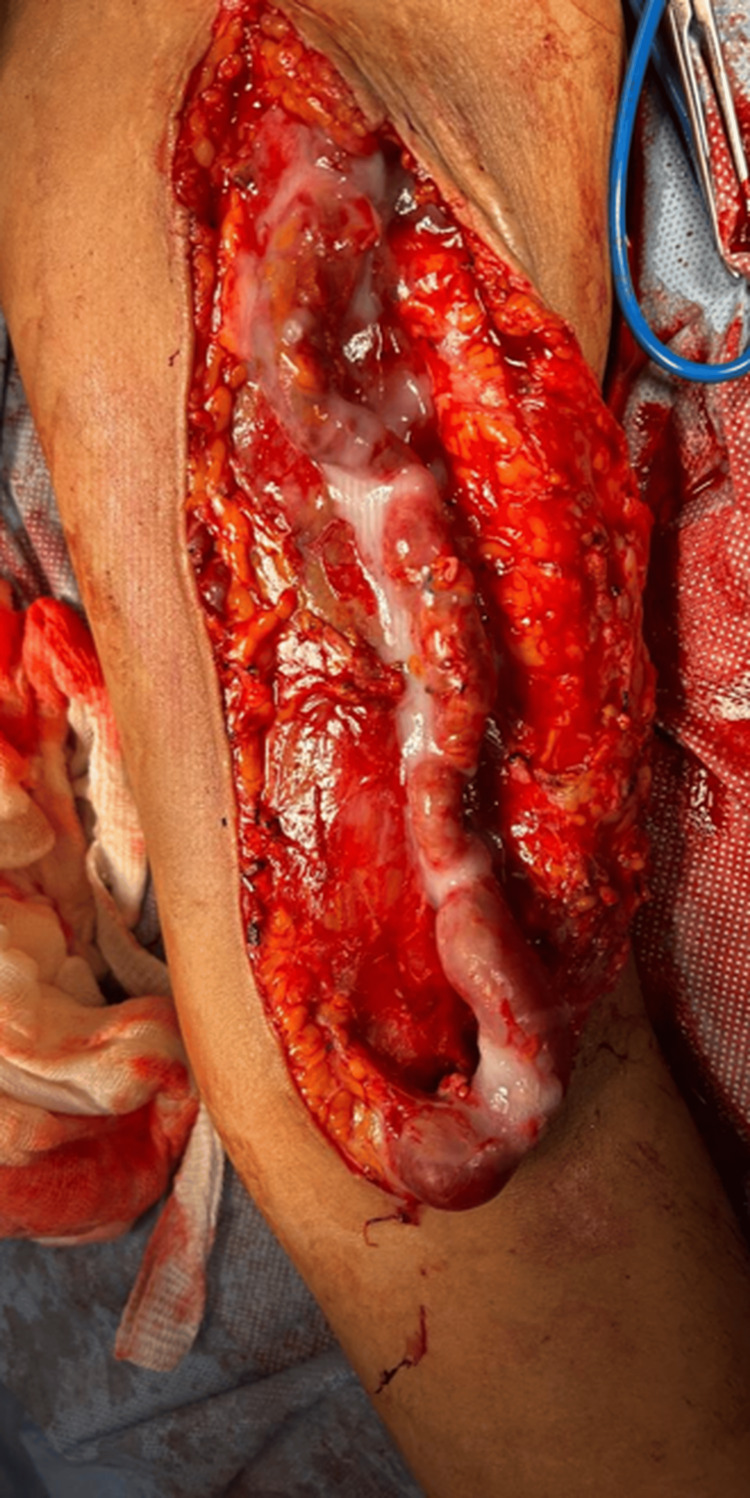
Anastomosis Appropriate end-to-end vein-to-vein anastomosis with fibrin sealant.

The proximal and distal venous segments were connected end-to-end using clamps. The bleeding was stopped with hemostatic stitches until a secure seal was established. Correct blood flow through the repaired vein was confirmed, and the wrist pulse was verified. The fibrin sealant was used to increase the tightness (Figure 5). The skin was close, and the thrill was confirmed.

The patient spent two days in the hospital without any complications. A significant decrease in pain was reported, and the patient was discharged. Follow-up ultrasound confirmed adequate flow two months after surgery, resulting in a decision to initiate hemodialysis.

## Discussion

Less than 8% of complications involving AVFs are due to aneurysm formation, which is defined as a dilatation of the venous segment that is twice the normal diameter or measures between 2 and 3 cm in diameter. The most common causes of aneurysm formation in AVFs include increased blood flow or fistula stenosis, as seen in our case, where the synechiae found inside the vein may have contributed to this issue [3-7].

**Figure 6:**
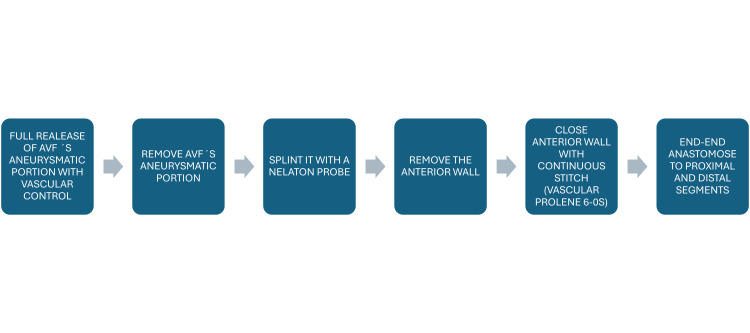
Procedure timeline The step-by-step procedure has been summarized in a timeline for better description. AVF: arteriovenous fistulas

Premature aneurysm development in our patient is associated with a significant risk factor: immunosuppressive treatment, which has been linked to an increased risk of aneurysm formation, particularly in patients who have undergone kidney transplants. The pathogenesis of aneurysmal degeneration in AVFs involves chronic turbulent flow, progressive venous wall dilation, and endothelial injury leading to intimal hyperplasia, mural thrombus formation, and perivascular fibrotic adhesions to surrounding tissues, which are frequently observed during surgical exploration [1,7]. Most of the literature recommends that the optimal time for treating aneurysms is when patients experience symptoms, with pain being the primary and most frequently reported symptom. Conservative management is acceptable in small, stable aneurysms, but intervention is indicated in cases of rapid enlargement, skin thinning, infection, or risk of rupture [1,7,8]. Further research is required to evaluate the various treatment options, allowing for individualized care and the selection of endovascular or surgical procedures, which may involve the use of autologous or prosthetic grafts or a primary repair, considering the benefits and drawbacks of each approach to maximize the patient`s outcomes and limit the risk of complications [9,10]. In our patient, the aneurysmatic portions in the venous segment were resected, and primary repair was performed (Figure 6), resulting in favorable outcomes.

## Conclusions

Arteriovenous fistulas can lead to aneurysms, which are often treated using various methods. Our case report aims to increase knowledge of the surgical approach and complement the literature with information on safe management, thereby supporting a recommended treatment choice and suggesting a paradigm for future comparisons of established treatments versus randomized studies.
